# Serum Trace-Element Profiles and Exploratory Composite Indices in Multiple System Atrophy: A Case–Control Study

**DOI:** 10.3390/diagnostics16152420

**Published:** 2026-07-31

**Authors:** Aslı Aksoy Gündoğdu, Bekir Enes Demiryürek

**Affiliations:** 1 Department of Neurology, Faculty of Medicine, Tekirdağ Namık Kemal University, Tekirdağ 59030, Turkey; aagundogdu@nku.edu.tr; 2 Department of Neurology, Faculty of Medicine, Istanbul Aydın University, Istanbul 34295, Turkey

**Keywords:** multiple system atrophy, trace elements, aluminum, copper, Metal Burden Index, ICP-MS, case–control study

## Abstract

**Background/Objectives:** Circulating trace-element differences have been reported in neurodegenerative disorders, but data in multiple system atrophy (MSA) are scarce. We compared eleven trace elements and two operationally defined exploratory composite indices between patients with MSA and controls. **Methods:** This case–control study included 30 patients with MSA and 30 frequency-matched controls. Eleven elements were quantified by ICP-MS. The raw Metal Burden Index (MBI; mean of aluminum, lead, cadmium, manganese, and arsenic) and Neurotoxic Metal Score (NMS) were calculated. Component-level decomposition was performed, and an aluminum-excluded index and component-standardized MBI (MBIz) were evaluated as sensitivity analyses. **Results:** Patients with MSA had higher aluminum and copper and lower iron and zinc than controls (all *p* < 0.001, Bonferroni-corrected; absolute Cohen’s d = 3.39–3.85). The raw MBI was higher in MSA (*p* < 0.001, d = 1.38) and remained associated with MSA status after age/sex adjustment (OR = 5.50, 95% CI 2.25–13.47 per 1-SD increase). Aluminum accounted for 114.6% of the MBI difference; the aluminum-excluded index did not differ. MBIz was higher in MSA (*p* < 0.001, d = 0.95). Exploratory ROC analyses showed apparent separation for raw MBI, aluminum, and copper without establishing validated diagnostic thresholds. No statistically significant correlations with disease severity or duration were observed; estimates were imprecise. **Conclusions:** MSA was associated with higher serum aluminum and copper and lower iron and zinc. The raw MBI predominantly reflected the aluminum signal; its incremental value beyond individual elements remains unproven. Findings require validation in disease-control and multicenter cohorts.

## 1. Introduction

Multiple system atrophy (MSA) is a rare, rapidly progressive, and fatal neurodegenerative disorder characterized by varying combinations of autonomic failure, parkinsonism, and cerebellar ataxia. Pathologically, it is classified as an α-synucleinopathy, defined by the accumulation of misfolded α-synuclein within oligodendroglial cytoplasmic inclusions [1,2,3]. The estimated prevalence ranges from approximately 3–5 cases per 100,000 individuals, with a median survival of 6–10 years following symptom onset [4]. Despite advances in clinical and neuropathological characterization, the mechanisms underlying disease onset and progression remain incompletely understood. In addition to α-synuclein aggregation, oxidative stress, mitochondrial dysfunction, and neuroinflammation are increasingly recognized as key contributors to neuronal damage in MSA [2,5].

Environmental factors have been increasingly implicated in the pathogenesis of neurodegenerative diseases, including exposure to toxic metals and other environmental pollutants [6]. Among these, disturbances in metal homeostasis have attracted considerable attention because of their potential to promote oxidative stress, protein misfolding, mitochondrial dysfunction, and neuroinflammatory responses [7,8,9]. Essential trace elements such as iron (Fe), copper (Cu), manganese (Mn), and zinc (Zn), as well as non-essential or toxic elements such as lead (Pb), cadmium (Cd), arsenic (As), and aluminum (Al), may influence neuronal vulnerability through distinct but overlapping biological pathways [7,8]. Alterations in elemental balance have been reported in several neurodegenerative disorders, including Parkinson’s disease and Alzheimer’s disease, where systemic and central changes have been linked to disease-related mechanisms [7].

Neuropathological and neuroimaging studies have consistently demonstrated abnormal iron accumulation within the basal ganglia in MSA, particularly in the putamen [2,5]. These findings are consistent with regional abnormalities in iron handling in MSA, although they do not establish systemic metal dysregulation. However, while brain iron deposition has been extensively investigated, considerably less is known about circulating trace-element profiles in patients with MSA [2,5].

Peripheral metal-related evidence in MSA remains limited and has largely focused on isolated Cu-related measures. In a small clinical series of 14 patients with MSA, hypoceruloplasminemia was identified in two patients; the same study also reported two autopsy-confirmed cases of MSA with a-/hypoceruloplasminemia [10]. A subsequent case report described an autopsy-confirmed patient with MSA, a heteroallelic ceruloplasmin-gene mutation, and reduced serum ceruloplasmin and Cu levels [11]. These reports involved small, clinically selected samples and did not systematically assess a broad range of circulating trace elements. To our knowledge, no directly comparable case–control study has evaluated a broad serum trace-element panel in MSA.

In recent years, increasing attention has been directed toward identifying accessible peripheral biomarkers that reflect underlying pathological processes in neurodegenerative disorders [2,12]. Serum-based biomarkers are particularly attractive because of their minimally invasive nature and potential applicability in clinical and research settings [12]. Circulating trace-element concentrations may provide complementary information on systemic trace-element status, and the simultaneous evaluation of multiple elements may characterize the serum concentration pattern more comprehensively than individual markers alone [7]. Moreover, the relationship between circulating trace-element concentrations and clinical severity in MSA has not been well established [2,5].

Therefore, the present study aimed to characterize serum trace-element profiles in patients with MSA compared with frequency-matched healthy controls and to explore their association with disease severity. In addition, two operationally defined exploratory composite indices summarizing selected serum metal concentrations were evaluated in MSA.

## 2. Materials and Methods

### 2.1. Study Design and Participants

This case–control study was conducted at the Department of Neurology of a tertiary university hospital. All patients met the 2022 Movement Disorder Society (MDS) diagnostic criteria [3] for clinically established MSA and were consecutively enrolled from the outpatient clinic. None of the included patients were classified as having clinically probable MSA. Healthy volunteers without known neurological or systemic diseases were included as the control group. Controls were frequency matched to the MSA group to achieve comparable age and sex distributions at the group level.

A total of 60 participants were enrolled, including 30 patients with MSA and 30 healthy controls. Participants were eligible if they were aged 40 years or older and provided written informed consent. The exclusion criteria applied to both groups included the presence of cardiological, respiratory, renal, hepatic, or neurological disorders other than MSA, intestinal malabsorption disorders, or active infection. Additional exclusion criteria included the use of thyroid hormones or lithium, psychotropic medications unrelated to MSA treatment, vitamin or mineral supplements, current or former smoking, alcohol abuse, known occupational or environmental metal exposure, and metallic implants such as prostheses or surgical screws. Antiparkinsonian and autonomic medications used for MSA management were permitted and recorded. Known occupational or environmental metal exposure was assessed through structured clinical interview and was used as an exclusion criterion.

The study protocol was approved by the Ethics Committee of Tekirdağ Namık Kemal University Faculty of Medicine (Approval No: 2026.122.04.16). The study was conducted in accordance with the Declaration of Helsinki, and written informed consent was obtained from all participants prior to inclusion.

### 2.2. Clinical Assessment

Demographic characteristics, including age and sex, were recorded for all participants. In patients with MSA, detailed clinical information was obtained, including disease duration and clinical subtype, classified as MSA with predominant parkinsonism (MSA-P) or MSA with predominant cerebellar features (MSA-C). Brain MRI was reviewed in all patients, and each had at least one characteristic finding supporting clinically established MSA and concordant with the predominant phenotype, including the putaminal rim sign in MSA-P and cerebellar atrophy and/or the hot cross bun sign in MSA-C.

Disease severity was evaluated using the Unified Multiple System Atrophy Rating Scale (UMSARS), a validated clinical instrument commonly used to assess functional impairment and disease progression in MSA [13]. UMSARS assessments were performed by a single neurologist experienced in movement disorders to reduce inter-rater variability. Both UMSARS Part I, which evaluates activities of daily living, and UMSARS Part II, which evaluates motor examination findings, were recorded. The total UMSARS score was calculated by summing the scores of both parts and was used as a measure of overall disease severity. Concomitant medications used at the time of blood sampling were recorded from clinical interviews and medical records. Levodopa-equivalent daily dose (LDED) was calculated for antiparkinsonian treatment. The use of levodopa, amantadine, rasagiline, fludrocortisone, dopamine agonists, midodrine, and droxidopa was documented. Antihypertensive use was not included in the exploratory medication analyses.

### 2.3. Blood Sampling and Laboratory Analysis

Venous blood samples were collected from all participants between 08:00 and 10:00 after an overnight fast of at least 8 h. Venous access was obtained using an indwelling intravenous cannula. The metallic trocar was used only for vein puncture and was withdrawn immediately after access was secured; blood was subsequently collected through the plastic catheter sheath to minimize contact with metallic surfaces. The first 2–3 mL of blood was discarded before collection of the study sample. Blood was then collected into VACUSERA^®^ Trace Element Serum Clot Activator Tubes (Disera Tıbbi Malzeme Lojistik Sanayi ve Ticaret A.Ş., İzmir, Türkiye; catalog no. 235006; 6.0 mL; 13 mm × 100 mm; serum clot activator; dark-blue closure) from a single manufacturing lot. Tourniquet application was kept brief, and all subsequent handling was performed using metal-free polypropylene materials; glassware and metal-containing processing materials were avoided.

Samples were kept upright at room temperature, allowed to clot for approximately 30 min, and centrifuged at 1500× *g* for 10 min at 4 °C; serum separation was completed within 60 min of collection. The separated serum was visually inspected for hemolysis, lipemia, and icterus. Hemolysis was also assessed quantitatively by measuring the hemolysis index on an Alinity c clinical chemistry analyzer (Abbott Laboratories, Abbott Park, IL, USA). Samples classified as hemolyzed according to the laboratory’s acceptance criteria, as well as those showing lipemia or icterus, were excluded. Serum was immediately transferred using trace-element–compatible pipette tips into trace-element–free polypropylene cryotubes. Aliquots were stored at −80 °C until analysis and were not subjected to repeated freeze–thaw cycles.

Serum concentrations of aluminum (Al), arsenic (As), cadmium (Cd), cobalt (Co), chromium (Cr), copper (Cu), iron (Fe), manganese (Mn), nickel (Ni), lead (Pb), and zinc (Zn) were measured by inductively coupled plasma mass spectrometry (ICP-MS) using an Agilent 7900 ICP-MS system (Agilent Technologies, Santa Clara, CA, USA) at Acıbadem Labmed Clinical Laboratories, İstanbul, Türkiye (TÜRKAK Accreditation No. AB-0001-TL). Analyses followed the laboratory’s validated ICP-MS method and standard operating procedure (LM-MET-TL-010).

Internal standards were used to correct for instrumental drift, signal suppression, and matrix effects. Multi-element calibration materials and certified quality-control materials were used to verify analytical accuracy, and quality-control results were required to remain within predefined acceptance ranges. Patient and control samples were analyzed in the same batch in randomized mixed order, with laboratory personnel blinded to case–control status. Duplicate measurements were performed for each sample, and the intra-assay coefficient of variation was below 7% for all elements. For Al, the laboratory-reported limit of detection was ≤0.5 µg/L and the limit of quantification was 1–2 µg/L. Concentrations were reported in µg/L.

Routine biochemical parameters potentially relevant to metal metabolism or systemic confounding were also recorded, including ferritin, C-reactive protein, creatinine, estimated glomerular filtration rate (eGFR), and albumin. eGFR was calculated using the 2021 race-free CKD-EPI creatinine equation. These parameters were measured using routine accredited clinical chemistry procedures.

### 2.4. Composite Metal Indices

Two operationally defined exploratory composite indices were calculated to summarize selected serum trace-element concentrations. The raw Metal Burden Index (MBI) was defined as the arithmetic mean of Al, Pb, Cd, Mn, and As concentrations, all expressed in µg/L. The Neurotoxic Metal Score (NMS) was defined as the sum of Pb, Cd, and Mn concentrations. These three elements were selected as a narrower exploratory grouping because they are commonly discussed as neurotoxic metals in the environmental and neurodegeneration literature [7,14]. The NMS was intended as a restricted comparator to the broader five-component MBI rather than as a comprehensive index of all potentially neurotoxic elements. The exclusion of Al and As should therefore not be interpreted as indicating that they lack neurotoxic relevance. Neither index is a biologically validated measure of cumulative exposure or systemic metal burden, and their components were not standardized before calculation.

Two additional composite formulations were evaluated as sensitivity analyses. An Al-excluded index was calculated as the arithmetic mean of Pb, Cd, Mn, and As. A component-standardized MBI (MBIz) was calculated as the arithmetic mean of the z-scores of Al, Pb, Cd, Mn, and As, thereby placing all five components on a common scale before averaging.

### 2.5. Statistical Analysis

An initial sample-size calculation was performed using G*Power software (version 3.1.9.7; Heinrich-Heine-Universität, Düsseldorf, Germany) as a pragmatic recruitment-planning estimate for a generic two-group comparison. Assuming a two-sided α level of 0.05, 80% power, a 1:1 allocation ratio, and a large effect size of Cohen’s d = 0.75, the estimated minimum sample size was 58 participants. No individual trace element was prospectively designated as the primary endpoint, and the assumed effect size was not derived from a directly comparable MSA cohort. Accordingly, this calculation should be regarded as a feasibility benchmark rather than as a confirmatory power calculation for any specific analyte. It did not account for the Bonferroni-adjusted significance threshold applied to the 11 individual metal comparisons.

Statistical analyses were performed using IBM SPSS Statistics version 25.0 (IBM Corp., Armonk, NY, USA). Additional ROC-related analyses, including bootstrap confidence intervals (CIs) for the area under the curve (AUC) and leave-one-out cross-validation (LOOCV), were performed using R software, version 4.5.3 (R Foundation for Statistical Computing, Vienna, Austria). Continuous variables were expressed as mean ± standard deviation (SD) or median (minimum–maximum), depending on data distribution, whereas categorical variables were presented as numbers and percentages. The normality of continuous variables was assessed using the Shapiro–Wilk test.

Comparisons between the MSA and control groups were performed using the independent-samples *t*-test for normally distributed variables with homogeneous variances, Welch’s *t*-test when the homogeneity-of-variance assumption was not met, or the Mann–Whitney U test for non-normally distributed variables. Categorical variables were compared using the chi-square test or Fisher’s exact test, as appropriate. Matching was implemented at the group level; therefore, unpaired group comparisons and unconditional multivariable logistic regression were applied. Routine biochemical parameters, including ferritin, CRP, creatinine, eGFR, and albumin, were compared between groups and evaluated as potential systemic or metabolic confounders. Given the limited sample size and the number of events, these variables were not included simultaneously in the adjusted regression models to avoid overfitting. Instead, the adjusted regression models were restricted to age, sex, and one composite index at a time, while biochemical variables were interpreted descriptively in relation to potential systemic confounding.

For the 11 individual metal comparisons, Bonferroni correction was applied to control for multiple testing, resulting in an adjusted significance threshold of *p* < 0.0045. Composite indices were interpreted separately as operationally defined exploratory measures. Associations between serum metal levels, composite indices, and clinical parameters, including disease duration and UMSARS scores, were assessed using Pearson or Spearman correlation coefficients, as appropriate. For the Spearman correlation analyses involving disease duration and UMSARS scores, 95% confidence intervals were estimated using percentile bootstrap resampling with 2000 resamples.

Within the MSA group, exploratory Spearman correlation analyses were performed between LDED and Al, Cu, Fe, Zn, and raw MBI. Al, Cu, Fe, Zn, and raw MBI were also compared between users and non-users of amantadine, fludrocortisone, rasagiline, and any adjunctive medication using the Mann–Whitney U test. These analyses were considered exploratory because of the small subgroup sizes. Bonferroni correction was applied to the LDED correlation analyses.

For the component-level decomposition of the between-group difference in the raw MBI, each element’s mean difference was divided by five, consistent with the arithmetic-mean definition of the index, and expressed as a percentage of the total observed difference. As sensitivity analyses, the Al-excluded index and MBIz were compared between groups, with Cohen’s d calculated for the corresponding differences. Pearson correlation coefficients were used to assess the relationships of raw-concentration MBI and MBIz with Al.

Raw-concentration MBI and MBIz were evaluated in separate multivariable binary logistic regression models adjusted for age and sex. MSA status was coded as 0 = control and 1 = MSA, and sex as female = 0 and male = 1. Raw-concentration MBI was standardized after calculation from the unstandardized component concentrations, whereas MBIz was derived by z-standardizing each component before averaging. Odds ratios were reported per 1-SD increase. Variables were entered simultaneously, and model fit was assessed using the omnibus likelihood-ratio test, Hosmer–Lemeshow goodness-of-fit test, Cox and Snell R^2^, Nagelkerke R^2^, and apparent classification accuracy.

Al and Cu were not included in the multivariable models owing to complete or near-complete group separation and the resulting risk of unstable estimates. These markers were evaluated instead in exploratory ROC and internal reproducibility analyses. ROC curve analyses were performed to evaluate the exploratory discrimination of Al, Cu, raw-concentration MBI, the Al-excluded index, and MBIz between patients with MSA and controls. The AUC, sensitivity, and specificity were calculated, and optimal cut-off values were determined using the Youden index. Given the near-complete separation observed for some markers, 95% CIs for AUC estimates were obtained by bootstrap resampling using 2000 resamples and the percentile method. LOOCV was performed as an internal reproducibility analysis and cannot substitute for independent external validation. For each LOOCV iteration, the optimal Youden cut-off was derived after excluding one participant and was then applied to the excluded participant. These ROC analyses were considered exploratory and were not intended to establish clinically validated diagnostic thresholds in the absence of disease-control and external validation cohorts.

All statistical tests were two-tailed, and a *p* value < 0.05 was considered statistically significant unless otherwise specified. Effect sizes were calculated using Cohen’s d with pooled standard deviations to quantify the magnitude of group differences. Negative Cohen’s d values indicated lower concentrations in the MSA group compared with controls.

## 3. Results

### 3.1. Participant Characteristics

The study included 60 participants, comprising 30 patients with MSA and 30 healthy controls frequency-matched to the MSA group by age and sex. The demographic, clinical, and routine biochemical characteristics of the study population are summarized in Table 1. The groups were comparable in age, sex distribution, ferritin, albumin, CRP, creatinine, eGFR, and BMI. Among patients with MSA, the mean age at symptom onset was 56.77 ± 8.32 years and the mean disease duration was 5.97 ± 1.69 years. Twenty-five patients (83.3%) had MSA-P and five (16.7%) had MSA-C. Mean UMSARS Part I, Part II, and total scores were 29.67 ± 6.94, 38.73 ± 7.05, and 68.40 ± 13.60, respectively. All patients with MSA were receiving levodopa, with a mean levodopa-equivalent daily dose of 576.7 ± 243.1 mg/day. Twenty-one patients (70.0%) were also receiving adjunctive treatment, most commonly amantadine, fludrocortisone, or rasagiline; detailed medication exposure is presented in Table S1.

### 3.2. Comparison of Serum Metal Levels and Composite Indices

**Figure 1:**
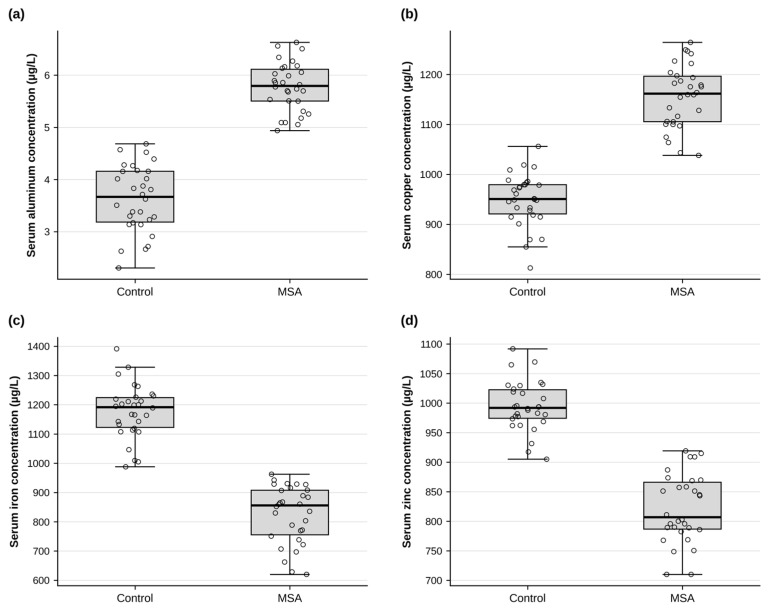
Distribution of serum aluminum (**a**), copper (**b**), iron (**c**), and zinc (**d**) concentrations in patients with multiple system atrophy and healthy controls. Boxes represent the interquartile range, horizontal lines indicate the median, and whiskers extend to the most extreme values within 1.5× the interquartile range. Individual participant observations (*n* = 30 per group) are superimposed as jittered open circles. All four between-group comparisons remained statistically significant after Bonferroni correction (*p* < 0.0045).

Serum metal concentrations and composite indices are presented in Table 2. Four of the 11 measured elements differed significantly between groups and survived Bonferroni correction. Compared with controls, patients with MSA had higher serum Al (5.78 ± 0.47 vs. 3.63 ± 0.64 µg/L; *p* < 0.001) and Cu (1156.17 ± 62.49 vs. 948.80 ± 52.33 µg/L; *p* < 0.001) (Figure 1), and lower Fe and Zn concentrations. Serum Fe was 825.09 ± 98.37 µg/L in the MSA group and 1176.17 ± 92.51 µg/L in controls (*p* < 0.001). Serum Zn was also lower in MSA than in controls (821.79 ± 58.59 vs. 995.02 ± 42.14 µg/L; *p* < 0.001).

Effect size analysis demonstrated very large group differences for Al (Cohen’s d = 3.85), Cu (d = 3.60), Fe (d = −3.68), and Zn (d = −3.39). As, Cd, Co, Cr, Mn, Ni, and Pb did not differ significantly between groups (all *p* > 0.05). Among the operationally defined exploratory composite indices, MBI was higher in patients with MSA than in controls (4.31 ± 0.26 vs. 3.94 ± 0.29; *p* < 0.001; d = 1.38), whereas NMS did not differ between groups (11.75 ± 1.05 vs. 11.87 ± 1.04; *p* = 0.658; d = −0.11).

### 3.3. Composite-Index Sensitivity and Multivariable Logistic Regression Analyses

Component-level decomposition showed that the between-group difference in the raw MBI was driven predominantly by Al. Al accounted for 114.6% of the observed raw MBI difference, whereas Pb, Cd, Mn, and As contributed −4.5%, 1.5%, −3.4%, and −8.2%, respectively. Together, the four non-Al components reduced the index difference by 0.055 µg/L. The Al-excluded index did not differ between patients with MSA and controls (3.946 ± 0.296 vs. 4.014 ± 0.332; *p* = 0.403; Cohen’s d = −0.22) and showed no meaningful discrimination (AUC = 0.424). By contrast, MBIz was higher in the MSA group (0.174 ± 0.370 vs. −0.174 ± 0.358; *p* < 0.001; Cohen’s d = 0.95) and showed moderate discrimination (AUC = 0.751). Its Pearson correlation with Al was lower than that of the raw-concentration MBI, although the Al influence was not eliminated (r = 0.458 vs. r = 0.652) (Table S2).

In age- and sex-adjusted logistic regression analyses, both composite formulations were associated with MSA status, with a larger effect estimate for the raw-concentration MBI than for MBIz (Table 3).

MSA status was coded as 0 = control and 1 = MSA. Sex was coded as female = 0 and male = 1. Raw-concentration MBI and MBIz were evaluated in separate multivariable binary logistic regression models adjusted for age and sex. Raw-concentration MBI was calculated as the arithmetic mean of the unstandardized serum concentrations of Al, Pb, Cd, Mn, and As and was subsequently standardized for regression analysis. MBIz was calculated by z-standardizing each component within the full sample and then averaging the standardized components. Odds ratios for both composite indices are reported per 1-SD increase. All variables within each model were entered simultaneously. Because of the case–control design, the reported odds ratios quantify associations with MSA status and should not be interpreted as estimates of disease risk. CI, confidence interval; MBI, Metal Burden Index; MBIz, component-standardized Metal Burden Index; MSA, multiple system atrophy; OR, odds ratio; SE, standard error.

Raw-concentration MBI was associated with MSA status after adjustment for age and sex (OR = 5.503 per 1-SD increase, 95% CI 2.249–13.467; *p* < 0.001), as was MBIz (OR = 2.901 per 1-SD increase, 95% CI 1.482–5.678; *p* = 0.002). Age and sex were not significantly associated with MSA status in either model. The Hosmer–Lemeshow test was non-significant for the raw-MBI model (*p* = 0.350) but significant for the MBIz model (*p* = 0.007), indicating possible calibration concerns for the latter. Al and Cu were not included in the multivariable models owing to near-complete group separation and were evaluated instead in exploratory ROC analyses.

### 3.4. Exploratory Correlation, ROC, and Internal Reproducibility Analyses

Across the 52 exploratory correlations between the 11 serum trace elements, the two main composite indices, and the four clinical measures, Spearman coefficients ranged from −0.346 to 0.348 (Table S3). The largest absolute estimates were observed between Fe and disease duration (ρ = 0.348, 95% CI −0.061 to 0.659; *p* = 0.060) and between Ni and disease duration (ρ = −0.346, 95% CI −0.684 to 0.063; *p* = 0.061). Neither association reached statistical significance, and both confidence intervals included zero. The remaining coefficients ranged from −0.273 to 0.234. Overall, no statistically significant correlations with disease duration or UMSARS scores were observed, although the generally wide confidence intervals indicate imprecision and do not exclude small-to-moderate associations. In exploratory within-MSA analyses, LDED was not correlated with Al, Fe, or raw MBI. A nominal inverse correlation was observed with Zn (ρ = −0.400, *p* = 0.028), but it did not remain significant after Bonferroni correction; the correlation with Cu was not significant (ρ = 0.344, *p* = 0.062). No significant differences in Al, Cu, Fe, Zn, or raw MBI were observed between users and non-users of adjunctive medications (minimum *p* = 0.061; Table S1). These analyses were limited by small subgroup sizes.

Exploratory ROC and internal reproducibility analyses are summarized in Table 4 and Figure S1. MBI showed moderate-to-good discrimination between patients with MSA and healthy controls (AUC = 0.833, bootstrap 95% CI 0.727–0.920). Cu and Al showed near-complete or complete separation in the present cohort, with AUC values of 0.998 and 1.000, respectively. LOOCV yielded high apparent accuracies for all three candidate markers. Given that cut-offs were derived within the same sample and the comparison was made against healthy rather than disease controls, these findings should be interpreted as exploratory and hypothesis-generating rather than as validated diagnostic thresholds.

## 4. Discussion

In this exploratory case–control study, we characterized serum trace-element profiles and two operationally defined exploratory composite indices in patients with MSA. The main findings were that patients with MSA showed a distinct serum trace-element pattern characterized by higher serum Al and Cu and lower Fe and Zn concentrations than frequency-matched healthy controls. In addition, MBI was higher in patients with MSA and remained associated with MSA status after adjustment for age and sex, whereas NMS did not differ between groups. Associations with disease duration and UMSARS-based severity were generally weak but imprecisely estimated. Exploratory ROC analyses showed apparent separation for MBI, Cu, and Al.

The combined findings indicate a serum trace-element pattern involving several elements rather than an isolated difference in a single element. Metal ions participate in oxidative stress, mitochondrial function, protein homeostasis, inflammatory signaling, and neuronal vulnerability, and interactions among Fe, Cu, Zn, Mn, and other elements have been described in neurodegenerative processes [7,8,14]. However, the present serum measurements cannot establish dysregulation of systemic metal homeostasis or disruption of these regulatory networks. Accordingly, the findings should be interpreted as concurrent differences in serum Al, Cu, Fe, and Zn concentrations rather than as evidence of a defined systemic regulatory disturbance.

The finding of reduced circulating Fe in patients with MSA should be considered in the context of neuroimaging and neuropathological evidence showing abnormal Fe accumulation in MSA, particularly in the putamen and other basal ganglia structures [2,5,15,16]. Quantitative susceptibility mapping studies have shown that regional susceptibility changes can help distinguish MSA from Parkinson’s disease and may reflect disease-related neuroanatomical vulnerability [15,16]. Our serum findings do not directly measure brain Fe deposition and should not be interpreted as a peripheral surrogate of central Fe accumulation. However, the coexistence of lower circulating Fe in the present cohort with previously reported regional brain Fe accumulation in MSA highlights a contrast between peripheral and central Fe findings [15,16,17]. The present study cannot establish altered systemic–central Fe handling, as serum and brain Fe were not assessed concurrently.

Cu was markedly elevated in patients with MSA. Cu is a redox-active metal involved in mitochondrial function, oxidative stress, and protein aggregation pathways [7,14]. Experimental studies indicate that Cu can interact with α-synuclein and influence aggregation kinetics, conformational transitions, and oxidative injury [18,19]. Given that MSA is an α-synucleinopathy characterized by oligodendroglial α-synuclein inclusions, elevated circulating Cu may be relevant to disease biology [2,3]. However, serum Cu cannot be assumed to reflect brain Cu accumulation or local Cu–α-synuclein interactions. Because circulating Cu is largely protein-bound, future studies should evaluate ceruloplasmin and non-ceruloplasmin-bound Cu to clarify whether the observed increase reflects altered bioavailable Cu or broader systemic changes. Therefore, the marked separation observed in our cohort requires replication and should be interpreted as a cross-sectional difference in serum Cu concentration rather than evidence of altered Cu homeostasis or a causal mechanism.

Al concentrations were also higher in the MSA group and showed complete separation from controls in exploratory ROC analysis. Al is a non-essential metal with recognized neurotoxic potential, and recent literature has linked Al exposure to oxidative stress, mitochondrial dysfunction, neuroinflammatory activation, epigenetic regulation, and impaired proteostasis [20,21,22]. These mechanisms overlap with pathways implicated in neurodegeneration and may provide biological plausibility for an association between increased Al and MSA-associated biological processes. At the same time, Al measurement is particularly vulnerable to pre-analytical contamination, and complete separation between groups is unusual for a circulating trace-element marker. Although the standardized pre-analytical and analytical procedures support measurement reliability, the complete separation observed for Al requires independent replication.

The reduced Zn concentrations observed in patients with MSA may also be biologically relevant. Zn is involved in synaptic transmission, antioxidant defense, immune regulation, and protein homeostasis [7,23]. Lower Zn may contribute to increased vulnerability to oxidative stress or reflect altered systemic nutritional, inflammatory, or metabolic regulation. In the present cohort, BMI, albumin, CRP, ferritin, creatinine, and eGFR did not differ between groups. However, disease-related alterations in dietary intake, swallowing, gastrointestinal function, hydration, physical activity, medication exposure, and unmeasured environmental factors were not comprehensively quantified and may still have contributed to the observed Zn difference.

Seven measured elements (As, Cd, Co, Cr, Mn, Ni, and Pb) did not differ between groups, arguing against a generalized shift across the serum trace-element panel. Exclusion of participants with known occupational or environmental metal exposure may have reduced variability in exposure-related elements such as Pb, Cd, and As. The absence of group differences in Pb, Cd, and Mn also provides a direct explanation for the null NMS result. Thus, the observed pattern was selective rather than a generalized increase in serum metal concentrations.

The raw MBI was operationally defined as an exploratory composite of five selected elements rather than as a biologically validated measure of cumulative exposure or systemic metal burden. Although the raw MBI was higher in patients with MSA and remained associated with MSA status after adjustment for age and sex, component-level analyses showed that the between-group difference was driven predominantly by Al. The four non-Al components collectively offset part of the Al contribution, and the Al-excluded index did not differ between groups. Component-wise standardization attenuated, but did not eliminate, the relationship with Al. Accordingly, the OR obtained for the raw MBI should be interpreted mainly as reflecting the Al signal carried by the composite rather than as evidence of an independent multi-element effect. MBI also showed lower discrimination than Al or Cu alone, and its incremental value beyond individual elements therefore remains unproven. MBIz should likewise be regarded as a sensitivity analysis rather than a validated alternative index, particularly in view of the potential calibration limitations observed in the adjusted model. Future studies should evaluate alternative approaches, including standardized composite scores, weighted indices, and data-driven multi-marker models, and should determine whether such approaches provide information beyond individual elements such as Al and Cu [7,12,14].

The lack of significant correlations between serum elements or composite indices and UMSARS scores suggests that the observed trace-element alterations were not clearly related to clinical severity in this cohort. This finding may have several explanations. First, circulating concentrations may not reflect regional brain metal deposition or cellular metal handling in the affected striatonigral and olivopontocerebellar systems. Second, MSA progression is driven by multiple mechanisms, including α-synuclein aggregation, glial dysfunction, neuroinflammation, mitochondrial impairment, and autonomic degeneration [2,5]. These processes may not be captured by serum trace-element levels alone. Third, the moderate sample size may have limited power to detect subtle clinical associations. Given the modest sample size and wide confidence intervals, small-to-moderate clinical associations cannot be excluded. The present study cannot determine whether these differences reflect MSA-associated processes, secondary disease-related factors, or other unmeasured influences.

The exploratory ROC findings require cautious interpretation. Al and Cu showed near-complete or complete separation, but all cut-offs were derived within the same small, single-center sample and against healthy controls. Accordingly, the reported cut-offs are apparent, hypothesis-generating estimates rather than clinically applicable diagnostic thresholds. Independent cohorts including Parkinson’s disease and other atypical parkinsonian disorders are needed to assess reproducibility and disease specificity [2,3]. Future studies should also determine whether trace-element profiles provide information beyond established imaging and fluid biomarkers [12,15,16].

This study has several strengths. First, it assessed a broad panel of eleven serum elements together with two operationally defined exploratory composite indices, enabling evaluation of both individual and integrated trace-element profiles. Second, the groups were frequency-matched by age and sex, and routine biochemical parameters including BMI, ferritin, albumin, CRP, creatinine, and eGFR were comparable between groups, reducing the likelihood that major differences in the measured nutritional, inflammatory, and renal parameters explained the findings. Third, the pre-analytical protocol was specifically designed for trace-element analysis, and measurements were performed by ICP-MS in an accredited laboratory, with same-batch randomized analysis, duplicate measurements, and blinded laboratory procedures. Fourth, the statistical approach incorporated multiple-testing correction, effect-size estimation, multivariable modeling, and bootstrap confidence intervals.

Several limitations should also be acknowledged. The study was single-center and included a relatively small sample. The marked predominance of MSA-P and the small number of patients with MSA-C precluded meaningful subtype-specific analyses; therefore, the findings should be considered primarily representative of MSA-P. The initial sample-size calculation was intended as a feasibility benchmark and did not account for the Bonferroni-adjusted threshold for the 11 metal comparisons, which may have limited power to detect smaller effects. The cross-sectional design precludes conclusions about causality, temporal sequence, or whether trace-element alterations precede disease onset. The absence of disease-control groups limits assessment of disease specificity and may have inflated the apparent discriminatory performance, particularly for Al and Cu. Serum measurements may not reflect central nervous system metal deposition or regional metal handling. Disease-related factors that may influence circulating trace-element concentrations, including altered dietary intake, dysphagia, gastrointestinal dysautonomia, altered intestinal transit, hydration status, and reduced physical activity, were not systematically quantified. All patients with MSA were receiving levodopa, precluding comparison with a levodopa-naïve group; moreover, the small subgroup sizes and uniform levodopa exposure do not exclude medication-related confounding, despite the absence of significant associations with LDED or adjunctive medication use. Antihypertensive medication use was not systematically assessed, so residual confounding cannot be excluded. Known occupational or environmental metal exposure was excluded through structured clinical interview; however, household water source, cookware materials, dietary metal content, and other chronic exposures were not quantified using a validated questionnaire. Ceruloplasmin-related Cu measures and a complete iron panel were also unavailable, limiting mechanistic interpretation of the Cu and Fe findings.

## 5. Conclusions

In this exploratory case–control study, patients with MSA showed a distinct serum trace-element profile characterized by higher serum Al and Cu and lower Fe and Zn concentrations. The raw MBI was higher in MSA and remained associated with MSA status after adjustment for age and sex; however, component-level analyses showed that this difference was driven predominantly by Al. The Al-excluded index did not differ between groups, and the incremental value of the raw MBI beyond individual elements remains unproven. The composite indices were operationally defined exploratory measures and should not be interpreted as validated constructs of systemic metal burden. No statistically significant associations with disease severity or duration were observed, although the estimates were imprecise and small-to-moderate associations cannot be excluded. Larger, multicenter, and longitudinal studies including Parkinson’s disease and other atypical parkinsonian syndromes are needed to assess reproducibility, disease specificity, and the biological significance of these serum differences.

## Figures and Tables

**Table 1:** Demographic, clinical, and routine biochemical characteristics of the study population.

Variable	MSA (*n* = 30)	Controls (*n* = 30)	*p* Value
Age, years	62.73 ± 8.67	62.93 ± 8.65	0.929
Sex, male/female	16/14	16/14	>0.999
BMI, kg/m^2^	25.76 ± 3.60	24.64 ± 2.80	0.182
Ferritin, ng/mL	238.72 ± 54.01	242.72 ± 55.87	0.779
Albumin, g/dL	4.20 ± 0.30	4.20 ± 0.27	1.000
CRP, mg/L	1.91 ± 1.02	2.28 ± 0.95	0.149
Creatinine, mg/dL	0.91 ± 0.21	0.91 ± 0.20	0.922
eGFR, mL/min/1.73 m^2^	83.6 ± 19.1	82.5 ± 16.0	0.814
Age at symptom onset, years	56.77 ± 8.32	—	—
Disease duration, years	5.97 ± 1.69	—	—
MSA subtype (MSA-P/MSA-C), *n* (%)	25/5 (83.3%/16.7%)	—	—
UMSARS Part I	29.67 ± 6.94	—	—
UMSARS Part II	38.73 ± 7.05	—	—
UMSARS Total	68.40 ± 13.60	—	—

Data are presented as mean ± SD or *n* (%). BMI: body mass index; CRP: C-reactive protein; eGFR: estimated glomerular filtration rate; MSA: multiple system atrophy; MSA-C: cerebellar-predominant MSA; MSA-P: parkinsonian-predominant MSA; UMSARS: Unified Multiple System Atrophy Rating Scale.

**Table 2:** Serum metal levels and composite indices in patients with MSA and controls.

Analyte	MSA (*n* = 30)	Controls (*n* = 30)	*p* Value	Cohen’s d
Al, µg/L	5.78 ± 0.47	3.63 ± 0.64	<0.001 *	3.85
As, µg/L	4.04 ± 0.77	4.19 ± 0.82	0.458	−0.19
Cd, µg/L	0.44 ± 0.07	0.41 ± 0.06	0.093	0.44
Co, µg/L	0.48 ± 0.08	0.47 ± 0.08	0.627	0.13
Cr, µg/L	0.48 ± 0.08	0.47 ± 0.09	0.574	0.11
Cu, µg/L	1156.17 ± 62.49	948.80 ± 52.33	<0.001 *	3.60
Fe, µg/L	825.09 ± 98.37	1176.17 ± 92.51	<0.001 *	−3.68
Mn, µg/L	8.88 ± 0.90	8.94 ± 1.03	0.797	−0.07
Ni, µg/L	0.98 ± 0.16	1.01 ± 0.19	0.419	−0.21
Pb, µg/L	2.44 ± 0.43	2.52 ± 0.41	0.439	−0.20
Zn, µg/L	821.79 ± 58.59	995.02 ± 42.14	<0.001 *	−3.39
Raw MBI	4.31 ± 0.26	3.94 ± 0.29	<0.001	1.38
NMS	11.75 ± 1.05	11.87 ± 1.04	0.658	−0.11

Values are presented as mean ± SD. Serum metal concentrations are expressed as µg/L. Group comparisons were performed using independent-samples *t*-test, Welch *t*-test, or Mann–Whitney U test, as appropriate. Bonferroni correction was applied to the 11 individual metal comparisons, with an adjusted significance threshold of *p* < 0.0045; asterisks denote comparisons that remained significant after correction. Composite indices were interpreted separately as operationally defined exploratory measures. Al: aluminum; As: arsenic; Cd: cadmium; Co: cobalt; Cr: chromium; Cu: copper; Fe: iron; Mn: manganese; Ni: nickel; Pb: lead; Zn: zinc; MBI: Metal Burden Index; NMS: Neurotoxic Metal Score.

**Table 3:** Age- and sex-adjusted logistic regression models evaluating the associations of raw-concentration MBI and component-standardized MBIz with MSA status.

Variable	*B*	SE	OR	95% CI for OR	*p* Value
Panel A. Model 1: Raw-concentration MBI					
Age	−0.003	0.039	0.997	0.924–1.075	0.930
Sex, male vs. female	0.047	0.662	1.048	0.286–3.837	0.943
Raw-concentration MBI, per 1-SD increase	1.705	0.457	5.503	2.249–13.467	<0.001
Constant	0.138	2.380	1.148	—	0.954
Panel B. Model 2: Component-standardized MBIz					
Age	0.002	0.035	1.002	0.935–1.074	0.945
Sex, male vs. female	0.024	0.603	1.025	0.314–3.343	0.968
MBIz, component-standardized, per 1-SD increase	1.065	0.343	2.901	1.482–5.678	0.002
Constant	−0.164	2.168	0.848	—	0.940

Model 1 summary: omnibus χ^2^ = 23.746, *df* = 3, *p* < 0.001; Hosmer–Lemeshow χ^2^ = 8.907, *df* = 8, *p* = 0.350; Cox and Snell R^2^ = 0.327; Nagelkerke R^2^ = 0.436; apparent classification accuracy = 70.0%. Model 2 summary: omnibus χ^2^ = 12.553, *df* = 3, *p* = 0.006; Hosmer–Lemeshow χ^2^ = 21.229, *df* = 8, *p* = 0.007; Cox and Snell R^2^ = 0.189; Nagelkerke R^2^ = 0.252; apparent classification accuracy = 68.3%. *B*: regression coefficient; CI: confidence interval; MBI: Metal Burden Index; MBIz: component-standardized Metal Burden Index; OR: odds ratio; SD: standard deviation; SE: standard error.

**Table 4:** Exploratory ROC and internal reproducibility analyses for candidate serum markers.

Marker	Cut-Off	AUC	95% CI	Sensitivity, %	Specificity, %	LOOCV Accuracy, %
MBI	>4.377	0.833	0.727–0.920	50.0	100.0	73.3
Cu, µg/L	>1037.9	0.998	0.990–1.000	100.0	96.7	96.7
Al, µg/L	>4.937	1.000	1.000–1.000	100.0	100.0	98.3

ROC analyses were performed to evaluate exploratory discrimination between patients with MSA and healthy controls. AUC 95% CIs were estimated by bootstrap resampling using 2000 resamples and the percentile method. Cut-off values were derived using the Youden index in the full sample and should therefore be considered apparent estimates. These values should not be interpreted as validated diagnostic thresholds in the absence of disease-control and external validation cohorts. AUC: area under the curve; CI: confidence interval; LOOCV: leave-one-out cross-validation; MBI: Metal Burden Index.

## Data Availability

De-identified participant-level data underlying the main analyses are provided in Dataset S1. Additional data may be made available from the corresponding author upon reasonable request, subject to institutional and ethical restrictions.

## Supplementary Materials

The following supporting information can be downloaded at: https://www.mdpi.com/article/10.3390/diagnostics16152420/s1, Figure S1: Receiver operating characteristic curves for raw MBI, Cu, and Al for discrimination between patients with MSA and healthy controls. AUC confidence intervals were estimated using percentile bootstrap resampling with 2000 resamples. Analyses were exploratory, and cut-offs were derived within the same case–control cohort; Table S1: Medication exposure in the MSA cohort and exploratory evaluation of medication use as a potential source of confounding; Table S2: Component-level contributions to the between-group difference in the raw Metal Burden Index and sensitivity analyses of alternative composite definitions; Table S3: Correlations between serum trace elements, composite indices, and clinical measures in patients with MSA; Dataset S1: De-identified participant-level dataset underlying the main analyses, with a separate variable dictionary worksheet.
