# Supplementary material for: Serum Trace-Element Profiles and Exploratory Composite Indices in Multiple System Atrophy: A Case–Control Study
Source: Diagnostics (Basel). 2026 Jul 31;16(15):2420. doi: 10.3390/diagnostics16152420 (PMC13465437; doi:10.3390/diagnostics16152420)
Supplement: Supplementary file 1 [file diagnostics-16-02420-s001.zip › diagnostics-4443273-supplementary.pdf]

Figure S1. Receiver operating characteristic curves for raw MBI, Cu, and Al for discrimination between patients with MSA and healthy controls. AUC confidence intervals were estimated using percentile bootstrap resampling with 2000 resamples. Analyses were exploratory, and cut-offs were derived within the same case–control cohort.

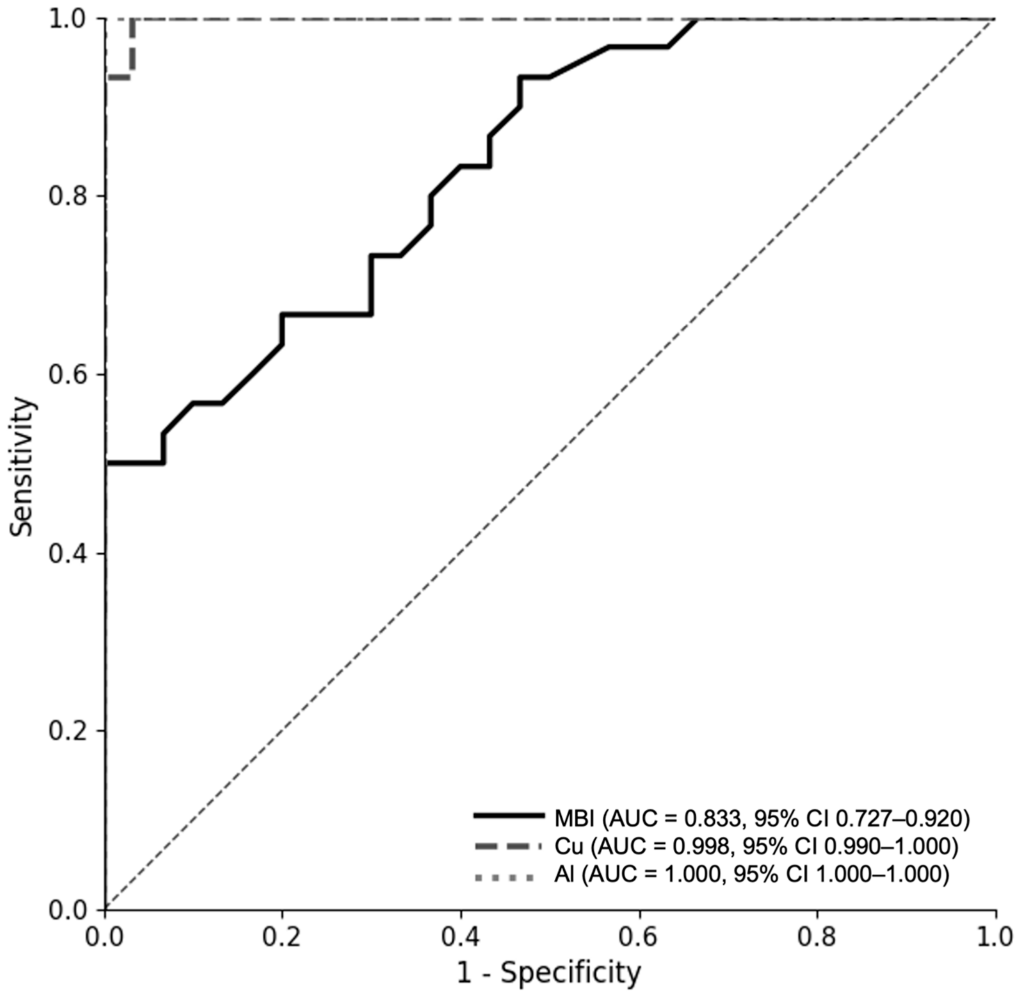

Table S1. Medication exposure in the MSA cohort and exploratory evaluation of medication use as a potential source of confounding

Panel A. Medication exposure in the MSA group (n = 30)

Medication	n (%)	Dose
Levodopa	30 (100.0)	LDED: 576.7 ± 243.1 mg/day; range, 200–1000
Amantadine	13 (43.3)	200–300 mg/day
Fludrocortisone	11 (36.7)	0.1 mg/day
Rasagiline	6 (20.0)	1 mg/day
Any adjunctive medication	21 (70.0)	—
Levodopa monotherapy	9 (30.0)	—

LDED, levodopa-equivalent daily dose. Antihypertensive medication exposure was not available in a sufficiently structured form for exploratory analysis. No control participant received any of the medications listed above.

Panel B. Serum trace-element concentrations and raw MBI according to adjunctive-medication use

Comparison, users vs. non-users	Al	Cu	Fe	Zn	Raw MBI
Amantadine, 13 vs. 17	0.950	0.427	0.586	0.357	0.933
Fludrocortisone, 11 vs. 19	0.796	1.000	0.606	0.061	0.263
Rasagiline, 6 vs. 24	0.756	0.667	1.000	0.351	0.667
Any adjunctive medication, 21 vs. 9	0.769	0.928	0.365	0.298	0.113

Values are p values from Mann–Whitney U tests comparing users and non-users. No comparison reached statistical significance; the smallest p value was 0.061 for Zn according to fludrocortisone use. These exploratory subgroup analyses were limited by small and unequal group sizes and therefore cannot exclude medication-related effects.

Panel C. Correlations of LDED with serum trace elements and raw MBI in the MSA group

Serum marker	Spearman's ρ	p value
Aluminum (Al)	0.080	0.673
Copper (Cu)	0.344	0.062
Iron (Fe)	−0.096	0.613
Zinc (Zn)	−0.400	0.028
Raw Metal Burden Index (MBI)	0.152	0.422

Correlations were evaluated within the MSA group using Spearman rank-correlation analysis. Bonferroni correction was applied for the five evaluated markers, yielding an adjusted significance threshold of $p < 0.010$. The inverse correlation between LDED and Zn was nominally significant but did not remain significant after correction for multiple comparisons. No other correlation reached nominal statistical significance. These exploratory findings do not exclude medication-related confounding.

Table S2. Component-level contributions to the between-group difference in the raw Metal Burden Index and sensitivity analyses of alternative composite definitions

Panel A. Component contributions to the between-group difference in the raw MBI

Component	Group mean difference (MSA − control), µg/L	Contribution to raw MBI difference	Percentage of total raw MBI difference
Aluminum (Al)	+2.150	+0.430	+114.6%
Lead (Pb)	−0.084	−0.017	−4.5%
Cadmium (Cd)	+0.029	+0.006	+1.5%
Manganese (Mn)	−0.064	−0.013	−3.4%
Arsenic (As)	−0.154	−0.031	−8.2%
**Total observed raw MBI difference**	—	**+0.375**	**100.0%**

The raw MBI was calculated as the arithmetic mean of the five raw serum concentrations. Each component's contribution was calculated as its between-group mean difference divided by five. Percentages were calculated relative to the total observed raw MBI difference. A contribution exceeding 100% indicates that the contributions of one or more other components were in the opposite direction, such that the four non-Al components collectively reduced the index difference by 0.055 µg/L.

Panel B. Sensitivity analyses of alternative composite definitions

Composite index	MSA, mean ± SD	Control, mean ± SD	*p* value	Cohen's d	AUC
Raw-concentration MBI	4.31 ± 0.26	3.94 ± 0.29	<0.001	1.38	0.833
Al-excluded index (Pb, Cd, Mn, and As)	3.95 ± 0.30	4.01 ± 0.33	0.403	−0.22	0.424
MBIz, component-standardized	0.17 ± 0.37	−0.17 ± 0.36	<0.001	0.95	0.751

The Al-excluded index was calculated as the arithmetic mean of Pb, Cd, Mn, and As. MBIz was calculated by z-standardizing Al, Pb, Cd, Mn, and As within the full sample and then averaging the five standardized components. The raw-concentration MBI and Al-excluded index were compared using independent-samples t-tests. For the Al-excluded index, a Mann–Whitney U sensitivity analysis also showed no evidence of a group difference ($p = 0.315$), consistent with the t-test result ($p = 0.403$). Higher index values were treated as indicating MSA in the ROC analyses.

Panel C. Correlations with Al and age- and sex-adjusted associations with MSA status

Composite index	Pearson r with Al	Adjusted OR for MSA per 1-SD increase (95% CI)	*p* value
Raw-concentration MBI	0.652	5.50 (2.25–13.47)	<0.001
MBIz, component-standardized	0.458	2.90 (1.48–5.68)	0.002

Each composite index was evaluated in a separate multivariable logistic regression model adjusted for age and sex. Odds ratios are reported per 1-SD increase in the respective index. The raw-concentration MBI was calculated from the arithmetic mean of the unstandardized component concentrations and was subsequently expressed per SD for regression analysis. MBIz was constructed by z-standardizing the individual components before averaging. **Abbreviations:** Al, aluminum; AUC, area under the receiver operating characteristic curve; Cd, cadmium; CI, confidence interval; MBI, Metal Burden Index; MBIz, component-standardized Metal Burden Index; Mn, manganese; MSA, multiple system atrophy; OR, odds ratio; Pb, lead; SD, standard deviation.

Table S3. Correlations between serum trace elements, composite indices, and clinical measures in patients with MSA

Marker	Clinical measure	Spearman's ρ	95% CI	*p* value
MBI	Disease duration	−0.019	−0.420 to 0.370	0.920
MBI	UMSARS Part I	0.059	−0.361 to 0.451	0.756
MBI	UMSARS Part II	0.101	−0.302 to 0.475	0.594
MBI	UMSARS Total	0.061	−0.330 to 0.455	0.749
NMS	Disease duration	−0.023	−0.399 to 0.360	0.905
NMS	UMSARS Part I	0.140	−0.263 to 0.499	0.461
NMS	UMSARS Part II	0.171	−0.195 to 0.519	0.365
NMS	UMSARS Total	0.147	−0.239 to 0.514	0.438
Al	Disease duration	−0.096	−0.464 to 0.296	0.613
Al	UMSARS Part I	−0.070	−0.450 to 0.299	0.713
Al	UMSARS Part II	−0.147	−0.543 to 0.259	0.440
Al	UMSARS Total	−0.143	−0.515 to 0.240	0.451
As	Disease duration	0.010	−0.363 to 0.379	0.958
As	UMSARS Part I	−0.049	−0.439 to 0.354	0.796
As	UMSARS Part II	−0.028	−0.386 to 0.344	0.882
As	UMSARS Total	−0.035	−0.419 to 0.352	0.855
Cd	Disease duration	0.139	−0.242 to 0.533	0.463
Cd	UMSARS Part I	0.026	−0.370 to 0.411	0.893
Cd	UMSARS Part II	0.115	−0.272 to 0.503	0.546
Cd	UMSARS Total	0.101	−0.291 to 0.492	0.594
Co	Disease duration	0.020	−0.335 to 0.377	0.915
Co	UMSARS Part I	−0.112	−0.467 to 0.257	0.557
Co	UMSARS Part II	−0.009	−0.384 to 0.363	0.960
Co	UMSARS Total	−0.041	−0.412 to 0.313	0.832
Cr	Disease duration	0.128	−0.247 to 0.469	0.500
Cr	UMSARS Part I	−0.028	−0.424 to 0.351	0.884
Cr	UMSARS Part II	0.112	−0.273 to 0.473	0.555
Cr	UMSARS Total	0.073	−0.348 to 0.451	0.700
Cu	Disease duration	0.127	−0.218 to 0.468	0.502
Cu	UMSARS Part I	0.156	−0.297 to 0.566	0.411
Cu	UMSARS Part II	0.157	−0.265 to 0.549	0.406
Cu	UMSARS Total	0.144	−0.291 to 0.546	0.447
Fe	Disease duration	0.348	−0.061 to 0.659	0.060
Fe	UMSARS Part I	−0.120	−0.477 to 0.265	0.527
Fe	UMSARS Part II	−0.110	−0.491 to 0.296	0.562
Fe	UMSARS Total	−0.102	−0.470 to 0.275	0.590
Mn	Disease duration	−0.090	−0.442 to 0.275	0.638
Mn	UMSARS Part I	0.103	−0.259 to 0.451	0.588
Mn	UMSARS Part II	0.137	−0.197 to 0.454	0.470
Mn	UMSARS Total	0.112	−0.226 to 0.451	0.556
Ni	Disease duration	−0.346	−0.684 to 0.063	0.061
Ni	UMSARS Part I	−0.273	−0.591 to 0.133	0.145
Ni	UMSARS Part II	−0.247	−0.590 to 0.166	0.188
Ni	UMSARS Total	−0.255	−0.574 to 0.130	0.173

Marker	Clinical measure	Spearman’s ρ	95% CI	*p* value
Pb	Disease duration	0.234	−0.167 to 0.594	0.213
Pb	UMSARS Part I	0.093	−0.300 to 0.440	0.625
Pb	UMSARS Part II	0.121	−0.258 to 0.474	0.524
Pb	UMSARS Total	0.084	−0.289 to 0.440	0.659
Zn	Disease duration	0.116	−0.276 to 0.499	0.542
Zn	UMSARS Part I	−0.039	−0.415 to 0.332	0.836
Zn	UMSARS Part II	0.013	−0.353 to 0.355	0.947
Zn	UMSARS Total	−0.011	−0.370 to 0.344	0.954

Values are Spearman’s ρ, percentile-bootstrap 95% confidence intervals based on 2000 resamples, and two-sided *p* values. Analyses were performed within the MSA group (n = 30). No correlation reached nominal statistical significance. All confidence intervals included zero, indicating substantial uncertainty and limited precision for small-to-moderate associations. MBI, Metal Burden Index; NMS, Neurotoxic Metal Score; Al, aluminum; As, arsenic; Cd, cadmium; Co, cobalt; Cr, chromium; Cu, copper; Fe, iron; Mn, manganese; Ni, nickel; Pb, lead; Zn, zinc; UMSARS, Unified Multiple System Atrophy Rating Scale.
